# MRI for adenomyosis: a pictorial review

**DOI:** 10.1007/s13244-017-0576-z

**Published:** 2017-10-04

**Authors:** Lisa Agostinho, Rita Cruz, Filipa Osório, João Alves, António Setúbal, Adalgisa Guerra

**Affiliations:** 1 Department of Radiology, Hospital Beatriz Angelo, Loures, Portugal; 2 Department of Gyneacology, Hospital da Luz, Lisbon, Portugal; 3 Department of Radiology, Hospital da Luz, Lisbon, Portugal

**Keywords:** Adenomyosis · Uterus · Female urogenital diseases · Magnetic resonance imaging · Diagnostic imaging

## Abstract

**Abstract:**

Adenomyosis is defined as the presence of ectopic endometrial glands and stroma within the myometrium. It is a disease of the inner myometrium and results from infiltration of the basal endometrium into the underlying myometrium. Transvaginal ultrasonography (TVUS) and magnetic resonance imaging (MRI) are the main radiologic tools for this condition. A thickness of the junctional zone of at least 12 mm is the most frequent MRI criterion in establishing the presence of adenomyosis. Adenomyosis can appear as a diffuse or focal form. Adenomyosis is often associated with hormone-dependent lesions such as leiomyoma, deep pelvic endometriosis and endometrial hyperplasia/polyps. Herein, we illustrate the MRI findings of adenomyosis and associated conditions, focusing on their imaging pitfalls.

***Teaching points*:**

• *Adenomyosis is defined as the presence of ectopic endometrium within the myometrium.*

• *MRI is an accurate tool for the diagnosis of adenomyosis and associated conditions.*

• *Adenomyosis can be diffuse or focal.*

• *The most established MRI finding is thickening of junctional zone exceeding 12 mm.*

• *High-signal intensity myometrial foci on T2- or T1-weighted images are also characteristic.*

## Introduction

Adenomyosis is a common benign gynaecological disorder defined as the presence of ectopic endometrial glands and stroma within the myometrium [1]. It is a disease of the archimetra or inner myometrium and results from infiltration of the basal endometrium into the underlying myometrium, with subsequent hypertrophy and hyperplasia of smooth muscle [2].

It is difficult to accurately determinate the incidence of adenomyosis since the diagnosis can only be made with certainty by microscopic examination of the uterus. Although generally estimated to affect 20% of women, the incidence was approximately 65% in one study in which meticulous histopathological analysis of multiple myometrial sections was performed [3].

The mean frequency of adenomyosis at hysterectomy is between 20% and 30% [4].

The aetiology of adenomyosis is still not fully understood and various theories have been proposed. Exposure to oestrogen [5], parity [5], and prior uterine surgery [6] are known risk factors. The most consensual theories propose that adenomyosis results from invagination of the endometrial basalis layer into the myometrium [7] or from embryologically misplaced pluripotent Müllerian remnants [8].

Histopathological examination allows direct visualisation of endometrial tissue inside the myometrium. Criteria for the histologic diagnosis of adenomyosis include the presence of penetrating glands at least: one low-power field from the endo-myometrial junction, 2.5 mm below the basal layer of endometrium or deeper than 25% of overall myometrial thickness [9]. Areas of myometrial smooth muscle proliferation are present around endometrial islands.

On gross pathology, the uterus is usually firm, enlarged and globular, with hypertrophic myometrial smooth muscle containing ectopic endometrium with dilated glands, cysts and haemorrhage.

So far, no study is available on the natural history of adenomyosis, and information regarding its prevalence and characteristics in adolescent girls and postmenopausal women remains limited. Some studies indicate that the diagnosis of adenomyosis is rare in adolescence [10] and features of classic adenomyosis are not typical [11], with cystic adenomyosis being almost specific to adolescent and young women [12]. The incidence of adenomyosis in adulthood significantly varies between studies, mainly because of differences in diagnostic criteria. In one study, the incidence varied from 12 to 58% between hospitals and 10–88% between pathologists [13]. In postmenopausal women, evidence points that adenomyosis begins during women’s fertile age [14].

Adenomyosis is asymptomatic in one third of cases, in the remaining being a cause of menorrhagia, dysmenorrhea, pelvic pain and uterine enlargement [15]. Its role in infertility is still debated, with a reported frequency of association of 1–14% [16]. There are, however, no large studies on this topic.

Clinical diagnosis of adenomyosis is usually difficult due to the nonspecific nature of symptoms and the confounding factor of coexistent pelvic diseases [17].

Transvaginal ultrasonography (TVUS) and magnetic resonance imaging (MRI) are the main radiologic tools for the diagnosis of adenomyosis [18]. MRI has a diagnostic accuracy of 85% [19], with added value in confirming the diagnosis and determining disease characteristics and extent and additional uterine lesions [20–22].

## MRI features

T2-weighted sequences are key for diagnosing adenomyosis since the sequences highlight the uterine zonal anatomy. T1-weighted imaging (T1-WI) also contributes to the diagnosis, by depicting high-signal intensity foci that represent haemorrhage. Gadolinium contrast enhancement does not aid in the diagnosis of diffuse adenomyosis [23], but should be considered in particular scenarios. Our protocol consists of pelvic T2-WI sagittal, axial and coronal planes and T1 3D fat-suppressed axial and sagittal planes. We use contrast when in doubt about the nature of a uterine nodule or to characterise associated findings, such as an adnexal mass.

Adenomyosis appears as increased thickness of the junctional zone, forming an ill-defined area of low signal intensity on T2, representing the smooth muscle hyperplasia accompanying the heterotopic endometrial tissue. This aspect is frequently associated with bright foci on T2-weighted images, which represent foci of heterotopic endometrial tissue, cystic dilatation of endometrial glands or haemorrhagic foci. If haemorrhagic, the foci are also bright on T1 FSWI images. This sign has the higher positive predictive value (95%) for the diagnosis of adenomyosis, however, with a low sensitivity (47.5%) [20].

Adenomyosis is mainly located in the fundus [20] and commonly observed in the posterior wall. The typical appearance is a large, rand asymmetric uterus, with a maximum junctional zone thickness of at least 12 mm and punctate high-intensity myometrial foci [17].

**Fig. 1:**
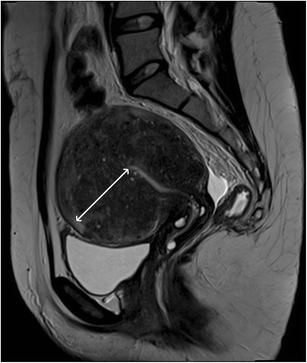
Diffuse adenomyosis: Sagittal T2-weighed image; thickening of the junctional zone forming an ill-defined area of low signal intensity, with punctate high-intensity myometrial foci (white arrow)

**Fig. 2:**
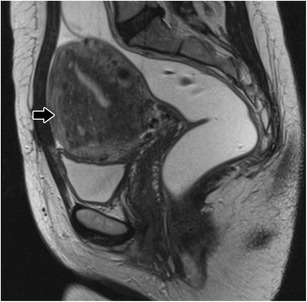
Focal adenomyosis: Sagittal T2-weighed image; focal asymmetric thickening of the junctional zone forming an ill-defined area of low signal intensity (black arrow)

There are two forms of adenomyosis: diffuse, in which foci of adenomyosis are distributed throughout the uterus (Fig. 1), and focal form, also named adenomyoma, when it affects a limited area (Fig. 2). The most frequent finding for the diagnosis of adenomyosis is thickening of the junctional zone, with a thickness exceeding 12 mm being highly predictive of the diagnosis [24, 25].

**Fig. 3:**
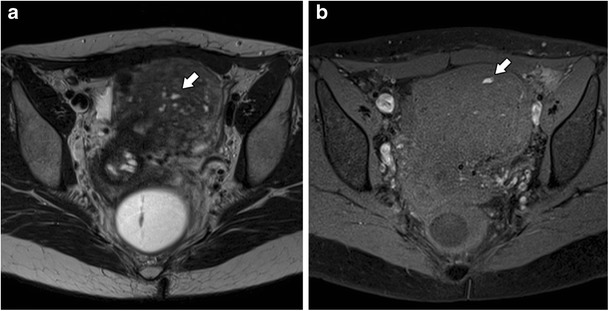
Focal adenomyosis: **a** Axial T2- and **b** Axial T1 3D FS-weighted images, showing embedded bright foci on T2- and T1 3D FS-weighted images representing haemorrhagic foci (white arrows)

A junctional zone thickness of less than 8 mm generally permits exclusion of the diagnosis [25, 26]. According to some authors, a junctional zone thickness between 8 and 12 mm can be diagnosed as adenomyosis, but requires ancillary criteria [22, 27]. These include a maximal junctional zone thickness to myometrium thickness ratio over 40% such as a relative thickening of the junctional zone in a localised area [27], and a difference between the maximum and the minimum thickness of the junctional zone in both anterior and posterior portions of the uterus of more than 5 mm [22]. One should also look for poorly defined limits of the junctional zone, the presence of high-signal intensity foci on T2- or T1-weighted sequences (Fig. 3) and linear striations of high T2 signal radiating from the endometrial zona basalis into the myometrium.

## Pitfalls in diagnosis

There are some pitfalls one should recognise when interpreting this type of examination, especially due to its frequent everyday occurrence. These pitfalls are mainly related to the menstrual phase effect on the junctional zone, postmenopausal condition, use of hormonal contraception and the presence of transient uterine contractions.

Thickness of the junctional zone is a hormone-dependent feature and changes according to the menstrual cycle. The uterus during menstruation may demonstrate marked thickening of the junctional zone, mimicking adenomyosis [19]. Preferably, MRI studies for adenomyosis should be performed in the late proliferative phase, avoiding the menstrual phase.

**Fig. 4:**
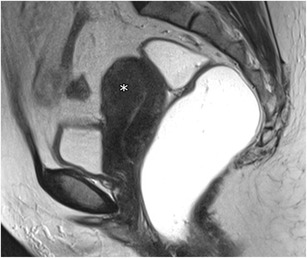
Postmenopausal uterus: Sagittal T2-weighted images; the junctional zone is not measurable (asterisk)

The junctional zone may not be measurable in approximately 30% of postmenopausal uteruses (Fig. 4) [20] and in women using contraceptive drugs, lowering the MRI sensitivity for the diagnosis.

**Fig. 5:**
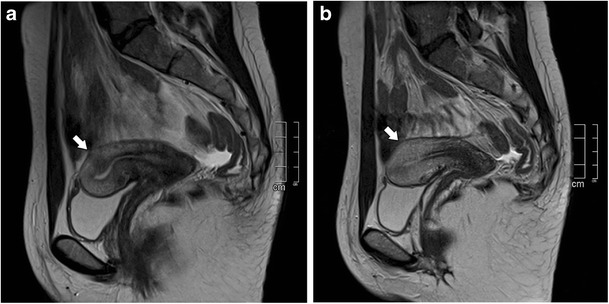
Uterine contractions mimicking adenomyosis: **a** and **b** Sagittal T2-weighted images; hypointense bands perpendicular to the junctional zone that modify after a few minutes (white arrows), and representing physiologic uterine contractions

Transient uterine contractions appear as T2-weighted hypointense bands perpendicular to the junctional zone or focal thickening of the junctional zone (Fig. 5), mimicking focal adenomyosis [28]. Repeating the acquisition of images within a few minutes may demonstrate their transient nature and help differentiate this physiologic process from adenomyosis. Administration of hyoscine may also be helpful [29].

**Fig. 6:**
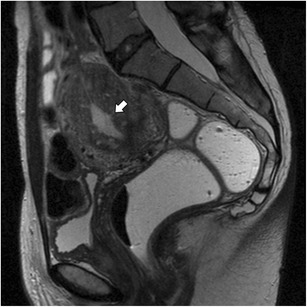
Pseudo-widening of the endometrium: Sagittal T2-weighted images; thickened junctional zone with striated high-signal intensity areas radiating from the endometrium toward the myometrium (white arrow), an appearance that simulates invasion by endometrial carcinoma

On the other hand, adenomyosis may mimic other pathologic conditions. An example is the so-called pseudo-widening of the endometrium (Fig. 6), a feature of adenomyosis that mimics endometrial carcinoma. Pseudo-widening of the endometrium represents an invasion of the myometrium by the basal endometrium and has a similar appearance to endometrial carcinoma invading the myometrium [17, 18].

## Unusual features of adenomyosis

### Adenomyoma and adenomyotic polyp

**Fig. 7:**
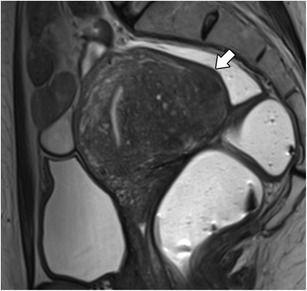
Adenomyoma: Sagittal T2-weighted image; circumscribed intra-myometrial hypointense mass with ill-defined margins and minimal mass effect with high-signal foci (white arrow)

**Fig. 8:**
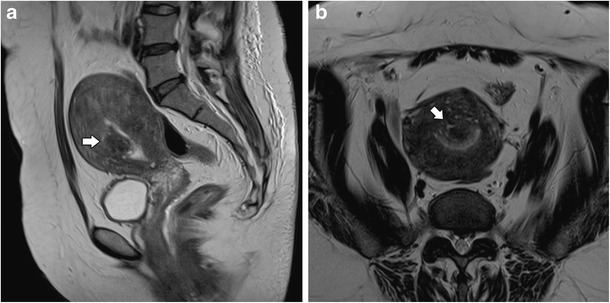
Polypoid adenomyoma: **a** Sagittal T2- and **b** Axial T2-weighted images; projection of junctional zone into the endometrial cavity with nodular morphology and ill-defined borders (white arrows)

An adenomyoma or focal adenomyosis (Fig. 7) represents a localised confluence of adenomyotic glands, constituting a mass-like form of adenomyosis [30]. It may appear as an intra-myometrial mass, most commonly situated in the corpus uteri. Occasionally, an adenomyoma bulges the endometrium, representing a submucosal adenomyoma. It can also protrude into the endometrium to grow as a polypoid mass, forming a polypoid adenomyoma (Fig. 8) [31]. Some authors distinguish between focal adenomyosis and adenomyoma, defining adenomyoma as a focal form of adenomyosis which is not in direct continuity with the junctional zone [32].

### Cystic adenomyosis

**Fig. 9:**
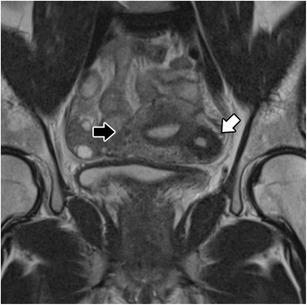
Isolated or juvenile cystic adenomyoma: Coronal T2-weighted images; nodular uterine lesion with a central cavity with hyperintense signal (white arrow), without connection to the endometrial cavity in an otherwise normal uterus (black arrow)

The cystic form of adenomyosis has mainly been reported in young women and is associated with severe medication-resistant dysmenorrhea, caused by extensive menstrual bleeding by the ectopic endometrium [14]. Histopathologic criteria for the diagnosis of an adenomyotic cyst include a cavity filled with haemorrhagic fluid that has no communication with the uterine cavity, is lined by endometrium and surrounded by myometrium [12]. It may be intramural, submucosal or subserosal. The cystic component (Fig. 9) appears with high-signal intensity on T1-weighted images and low signal on T2-weigted images, with surrounding adenomyotic tissue.

### Swiss cheese appearance

**Fig. 10:**
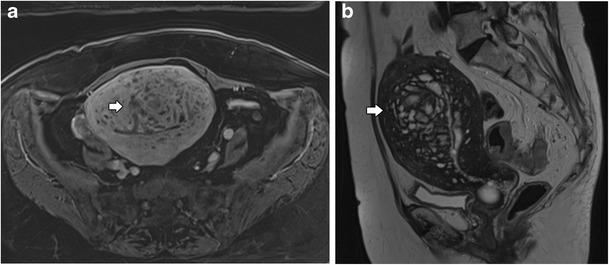
Swiss cheese appearance in adenomyosis: **a** Axial T1 3D FS- and **b** Sagittal T2-weighted images; poor definition of the endometrial junctional zone with exuberant glandular myometrial cysts, myometrial nodules and linear striations (white arrows)

This is a type of diffuse adenomyosis that may appear as a “Swiss cheese” appearance, with exuberant myometrial cysts and nodules on contrast-enhanced and T2 sequences. This “Swiss cheese” appearance is secondary to cross-sectional imaging of dilated endometrial glands within the myometrium [33]. With a Swiss cheese appearance, there is also widening and poor definition of the junctional zone and linear striations (Fig. 10).

## Differential diagnosis

### Leiomyoma

**Fig. 11:**
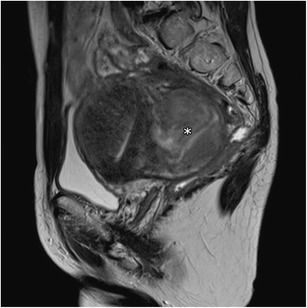
Leiomyoma: Sagittal T2-weighted image; heterogeneous and hypointense mass with well-defined borders, with mass effect on adjacent tissues (asterisk) representing leiomyoma. There are also features suggestive of adenomyosis

The main differential diagnosis of adenomyoma is leiomyoma. Adenomyoma appears as a hypointense mass on T2- weighted images with ill-defined borders, minimal mass effect and, in some cases, with multiple bright foci (Fig. 7). In counterpoint, leiomyomas mostly have well-defined borders, despite also being hypointense on T2-weighted images (Fig. 11). The presence of large vessels at the periphery may also favour this diagnosis [18].

### Acum

An isolated or juvenile adenomyotic cyst can be difficult to differentiate from a non-communicating accessory uterine cavity [34]. In fact, all these may have similar symptoms and imaging findings and may represent the same pathology—an accessory cavitated uterine mass (ACUM) with a functional endometrium [35]. It has been proposed that ACUMs represent a variety of the Mullerian anomaly generally located at the insertion of the round ligament [34]. They appear as a cyst with chocolate content lined by functional endometrium in histopathological examination. On MRI, they appear as a nodular uterine lesion with a central cavity with a hyperintense signal on T1-weighted images not connected to the endometrial cavity (Fig. 9). To establish the diagnosis, one may seek for an isolated accessory cavitated mass on MRI in an otherwise normal uterus. The differential diagnosis is broad, including rudimentary or cavitated uterine horns, adenomyosis with degenerated areas and degenerated leiomyomas [34].

## Associated conditions

**Fig. 12:**
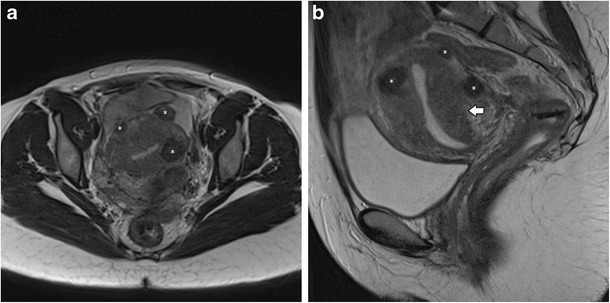
Diffuse adenomyosis and leiomyomas: **a** Axial T2 and **b** Sagittal T2-weighted images; diffuse thickening of the junctional zone (white arrow) in relation to diffuse adenomyosis and multiple hypointense masses representing leiomyomas (asterisks)

**Fig. 13:**
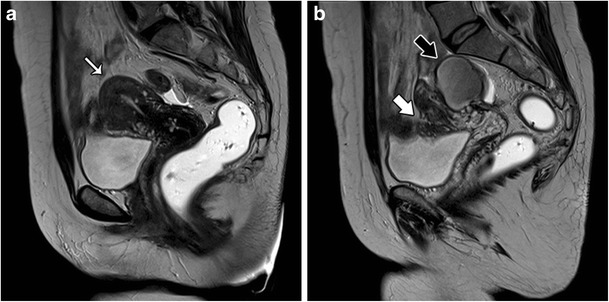
Adenomyosis and endometriosis: **a** and **b** Sagittal T2-weighted images; broadened junctional zone forming an ill-defined area of low signal intensity, with punctate high-intensity myometrial foci indicating adenomyosis (thin white arrow); endometriotic nodule in the bladder wall (white arrow); endometrioma in the left ovary (black arrow)

Adenomyosis is frequently associated with hormone-dependent pelvic lesions. Leiomyomas are present in almost 50% of cases involving adenomyosis of the uterus (Fig. 12) [19], while one third of young women with clinically suspected, deeply infiltrating endometriosis had MRI features of uterine adenomyosis [24] (Fig. 13). Adenomyosis also seems to be correlated with severe endometriosis [36].

**Fig. 14:**
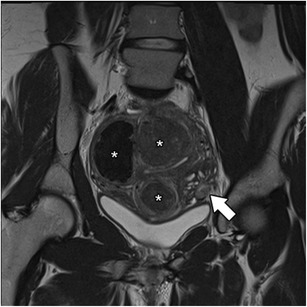
Subserosal endometriosis: Coronal T2-weighted image; subserosal ill-defined mass of low signal intensity, with high-intensity myometrial foci, in the left uterine wall from pelvic deep endometriosis. Leiomyomas are also seen (asterisks)

In the presence of an adenomyosis-like lesion in the subserosal region of the uterus, one should consider the hypothesis of subserosal endometriosis, with myometrial involvement (Fig. 14) [30]. The distinction is important, since the two conditions have different physiopathology—in invasive deep endometriosis, the lesion originates from outside the uterus and secondarily involves the serosal surface and outer myometrium. In this scenario, other findings of endometriosis should be sought.

**Fig. 15:**
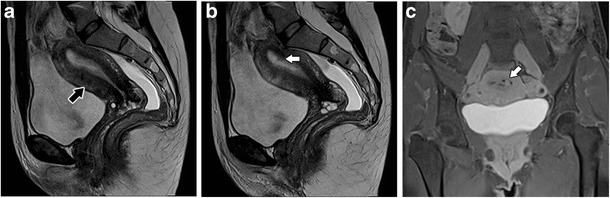
Diffuse adenomyosis and endometrial polyps: **a** and **b** Sagittal T2- and **c** Coronal contrast-enhanced T1 3D FS-weighted imagens; ill-defined thickening of the junctional zone in relation to adenomyosis (black arrow) and hypointense nodular formations in the endometrial cavity representing small endometrial polyps (white arrows)

Adenomyosis is a significant factor of sterility in these patients, presumably by impairing uterine sperm transport [37]. Adenomyosis is also associated with endometrial and cervical polyps (Fig. 15) [38].

## Conclusion

MRI represents an accurate evaluation tool for adenomyosis, allowing its diagnosis and detection of associated pathologies. It is important to recognise the usual and unusual characteristics of adenomyosis and be aware of pitfalls in order to make a correct diagnosis.
